# Morphological analysis of posterior malleolar fractures with intra-articular impacted fragment in computed tomography scans

**DOI:** 10.1186/s10195-021-00615-6

**Published:** 2021-12-10

**Authors:** Wenyong Xie, Hao Lu, Hailin Xu, Yuan Quan, Yijun Liu, Zhongguo Fu, Dianying Zhang, Baoguo Jiang

**Affiliations:** 1 Department of Orthopedics and Trauma, Peking University People’s Hospital, Beijing, 100044 China; 2 Trauma Center, National Center for Trauma Medicine, Beijing, 100044 China; 3 Key Laboratory of Trauma and Neural Regeneration, Peking University People’s Hospital, Beijing, 100044 China

**Keywords:** Posterior malleolus, Ankle fractures, Intraarticular fractures, Impact

## Abstract

**Background:**

Intraarticular impacted fragment (IAIF) of posterior malleolar fractures has been reported by a few studies. However its location, morphology, and the correlation of posterior malleolar fractures have not been described in detail. The aim of this study was to describe the morphology of IAIF in posterior malleolar fractures, to analyze the related factors between IAIF and posterior malleolar fragments, and explore the treatment of IAIF.

**Materials and methods:**

Between January 2013 and December 2018, 108 consecutive patients with unilateral posterior malleolar fractures were managed in our hospital. Basic demographic and computed tomography (CT) data were collected and classified by Lauge–Hansen, OTA/AO, Haraguchi, and Mason classification. Additional radiographic data, including the length and area of posterior malleolar fragment, IAIF, and stable tibial plafond were measured. The location of IAIF was described, and involvement of the fibular notch and medial malleolus was also observed. Statistics were analyzed based on univariate analysis (Chi-square test, *t*-test, Mann–Whitney *U* test, Fisher’s test) and Spearman’s correlation test.

**Results:**

Among the 108 cases of posterior malleolar fractures, 75 (69.4%) were with IAIF and 33 (30.6%) cases were without. There were 74 (68.5%) females and 34 (31.5%) males, and the average age of the patients was 49 years (18–89 years). The average *L*_IFN_/(*L*_IFN_ + *L*_SFN_) [length of involving fibular notch/(length of involving fibular + length of stable notch fibular notch)] was 32.9% (11.6–64.9%). The *A*_PMF_/(*A*_PMF_ + *A*_STP_ + *A*_IAIF_) [area of posterior malleolar fragment/(area of posterior malleolar fragment + area of IAIF + area of stable tibial plafond)] and *A*_IAIF_/*A*_PMF_ (area of IAIF/area of posterior malleolar fragment) were 13.1% (0.8–39.7%) and 52.6% (1.2–235.4%), respectively. Involvement of medial malleolus (fracture line extended to medial malleolus, *P* = 0.022), involvement of fibular notch (*P* = 0.021), *L*_IFN_/(*L*_IFN_ + *L*_SFN_) (*P* = 0.037), *L*_MPMF_ (*P* = 0.004), and *A*_PMF_ were significantly related to the occurrence of IAIF.

**Conclusion:**

Our research indicates a high incidence of IAIF in posterior malleolar fractures. All IAIFs were found in posterior malleolar, and the most common location was within the lateral area A. Posterior malleolar fracture lines that extend to medial malleolus or fibular notch herald the incidence of IAIF. *L*_IFN_/(*L*_IFN_ + *L*_SFN_), *L*_MPMF_ and *A*_PMF_ are also associated with the incidence of IAIF. CT scans are useful for posterior malleolar fractures to determine the occurrence of IAIF and make operational plans. Operation approach selection should be based on the morphology of posterior malleolar fragments and the location of IAIF.

**Level of evidence:**

Level III, retrospective case analysis.

## Introduction

Ankle fractures are commonly encountered, with 112–187 cases per 100,000 people reported per year [1, 2]. More than 40% of ankle fractures involve the posterior malleolus [3]. The classification, surgical indication, and fixation method of posterior malleolar fractures are the subject of increasing attention, but there is still no consensus on the treatment of posterior malleolar fractures [4–9]. The quality of reduction and fixation of posterior malleolar fractures is significant to the outcome of ankle fractures [9, 10].

Intraarticular impacted fragment (IAIF) in posterior malleolus could sometimes be identified on computed tomography (CT) scans of ankle fractures [11]. IAIF was first described in distal radius fractures, namely die-punch fragment [12], which was also common in pilon fractures [6]. Recently, Sultan et al. [13] described the characteristics of intercalary fragment as the IAIF in posterior malleolar fractures, and classified as free, folded, and compressed. IAIF is a kind of articular surface fragment resulting from impact and compressive forces. IAIF of posterior malleolar fractures has been reported in a few studies, with worse treatment outcomes predicted with IAIF [8–11, 14]. However, the location, morphology, and the correlation of ankle and posterior malleolar fractures classification of IAIF have not been described in detail.

The primary aim of this study is to describe the incidence, location, and morphology of IAIF of posterior malleolar fractures, and explore the related factors among IAIF and posterior malleolar fragments and fracture classifications.

## Methods

### Patient cohort

Institutional review board approval was obtained prior to initiation of this study.

We retrospectively analyzed 256 consecutive patients with ankle fractures from January 2013 and December 2018 at our institution. The inclusion criteria included the following: (1) patients had OTA/AO Type 44 fracture involving posterior malleolus [15], (2) patients had preoperative CT scans, and (3) patients underwent surgical treatment. Patients with (1) previous ankle surgery, (2) pathological fractures, (3) CT image with a slice thickness > 1 mm, and (4) age ≤ 16 years old were excluded.

### Demography

A total of 108 patients were finally included in this study. The average age was 49 years (18–89 years). There were 74 (68.5%) females and 34 (31.5%) males. The right ankle was more commonly involved than the left [63 (58.3%) right, 45 (41.7%) left]. The ankle fractures were classified according to the Lauge–Hansen and OTA/AO classification systems [16, 17]. The posterior malleolar fractures were classified according to the Haraguchi and Mason classification systems [18, 19] (Table 1).

**Table 1:** Patient characteristics (*N* = 108)

Patient characteristics	Overall number (%)	IAIF group number (%)	NIAIF group number (%)
Age (years)*	49 (18–89)	49 (22–89)	49 (18–89)
Sex^a^			
Male	34	20	14
Female	74	55	19
Injury site^a^			
Left	45	32	13
Right	63	43	20
Lauge–Hansen classification^a^			
Pronation-abduction	8	4 (50)	4 (50)
Pronation-external rotation	15	11 (73.3)	4 (26.7)
Supination-external rotation	85	60 (70.6)	25 (29.4)
OTA/AO classification^a^			
B	86	61 (70.9)	25 (29.1)
C	15	10 (66.7)	5 (33.3)
Haraguchi classification^a^			
1	71	47 (66.2)	24 (33.8)
2	25	22 (88)	3 (12)
3	12	6 (50)	6 (50)
Mason classification^a^			
1	12	6 (50)	6 (50)
2A	71	47 (66.2)	24 (33.8)
2B	22	20 (90.9)	2 (9.1)
3	3	2 (66.7)	1 (33.3)

*IAIF* intraarticular impacted fragments, *NIAIF* no intraarticular impacted fragments

^*^Values are given as the mean, with the range in parentheses

^a^Percentage was the ratio between the two groups

### Image analysis

CT data were loaded into Mimics software (V20.0, Materialize), in which the data measurements were made, such as length of the involving fibular notch, length of IAIF, length of major posterior malleolar fragments, displaced height of IAIF from articular surface, as well as area of IAIF, posterior malleolar fragments, and the stable plafond. All CT data were evaluated and measured by one experienced orthopedic doctor.

**Fig. 1:**
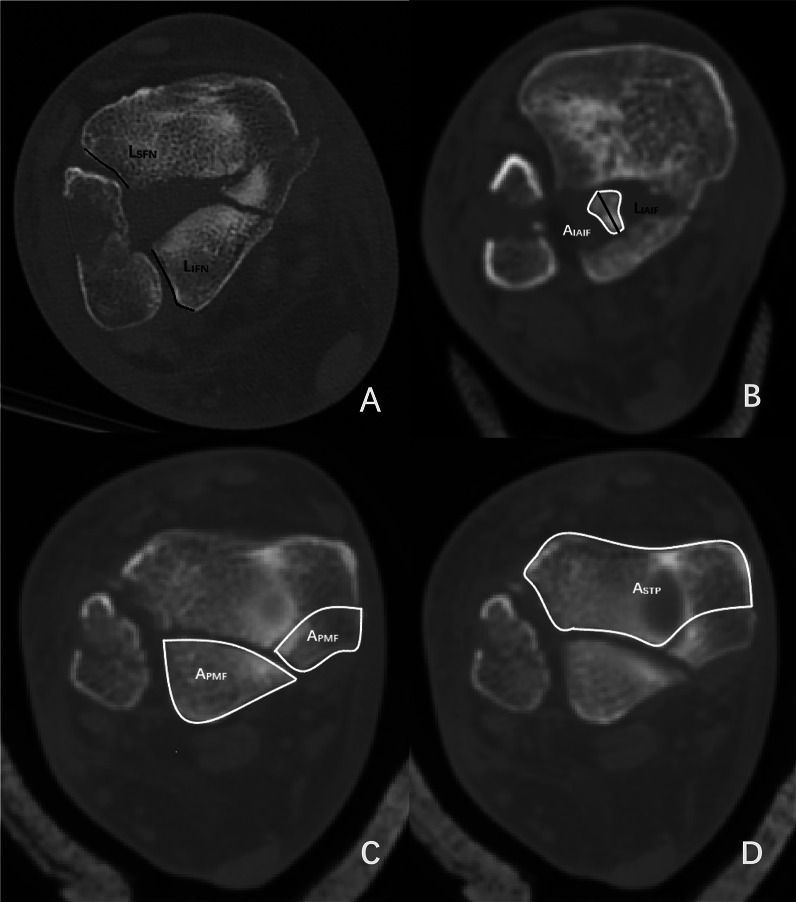
Example axial CT cross-sectional measurements. **A** Measured as the length of involved fibular notch (*L*_IFN_, length of involved fibular notch) and the stable fibular notch (*L*_SFN_, length of stable fibular notch). **B** Measured as the length and area of IAIF (*L*_IAIF_, length of IAIF, black; *A*_IAIF_, area of IAIF, white). **C** Measured as the area of posterior malleolar fragment (*A*_PMF_, area of posterior malleolar fragment). **D** Measured as the area of stable tibial plafond (*A*_STP_, area of stable tibial plafond)

On the axial CT cut of the tibial plafond, cross-sectional length and area were measured (Fig. 1): (1) tibiofibular syndesmosis axial (from tibial articular surface)—measured as the cross-sectional length of involved fibular notch (*L*_IFN_, length of involved fibular notch) and the stable fibular notch (*L*_SFN_, length of stable fibular notch); (2) IAIF articular surface axial—measured as the cross-sectional length and area of IAIF (*L*_IAIF_, length of IAIF; *A*_IAIF_, area of IAIF); (3) posterior malleolar fragment axial—measured as the cross-sectional area of posterior malleolar fragment (*A*_PMF_, area of posterior malleolar fragment); (4) stable plafond axial—measured as the cross-sectional area of stable tibial plafond (*A*_STP_, area of stable tibial plafond).

**Fig. 2:**
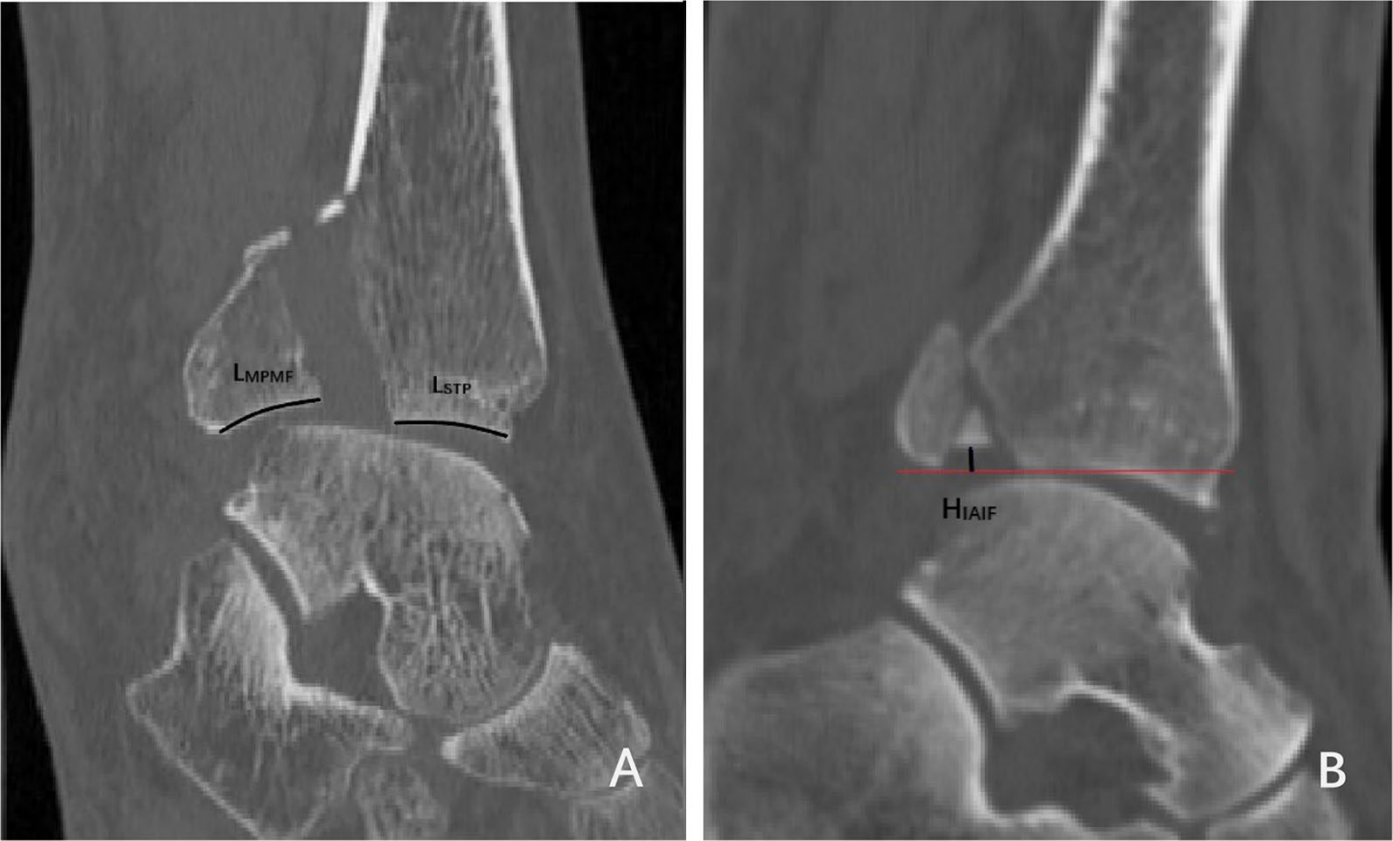
Example sagittal CT linear measurements. **A** Measured as the length of major posterior malleolar fragment (*L*_MPMF_, length of major posterior malleolar fragment) and stable tibial plafond (*L*_STP_, length of stable tibial plafond). **B** Measured as the displaced height of IAIF (*H*_IAIF_, height of IAIF) and the red line is the horizontal line of articular surface

On the sagittal CT cut of the tibial plafond, length measurements were made (Fig. 2): (1) major posterior malleolar fragment sagittal—measured as the distance from the anterior edge to the posterior edge of major posterior malleolar fragment (*L*_MPMF_, length of major posterior malleolar fragment); (2) tibial plafond sagittal—measured as the distance from the anterior edge of tibial plafond to the posterior edge of stable tibial plafond (*L*_STP_, length of stable tibial plafond); (3) IAIF sagittal—measured as the displaced height of IAIF from articular surface (*H*_IAIF_, height of IAIF).

*L*_IFN_/(*L*_IFN_ + *L*_SFN_), *A*_IAIF_/(*A*_PMF_ + *A*_STP_ + *A*_IAIF_), *A*_IAIF_/*A*_PMF_, *A*_PMF_/(*A*_PMF_ + *A*_STP_ + *A*_IAIF_) and *L*_MPMF_/(*L*_MPMF_ + *L*_STP_) were calculated. Whether posterior malleolar fractures were involved in the fibular notch and medial malleolus was observed.

**Fig. 3:**
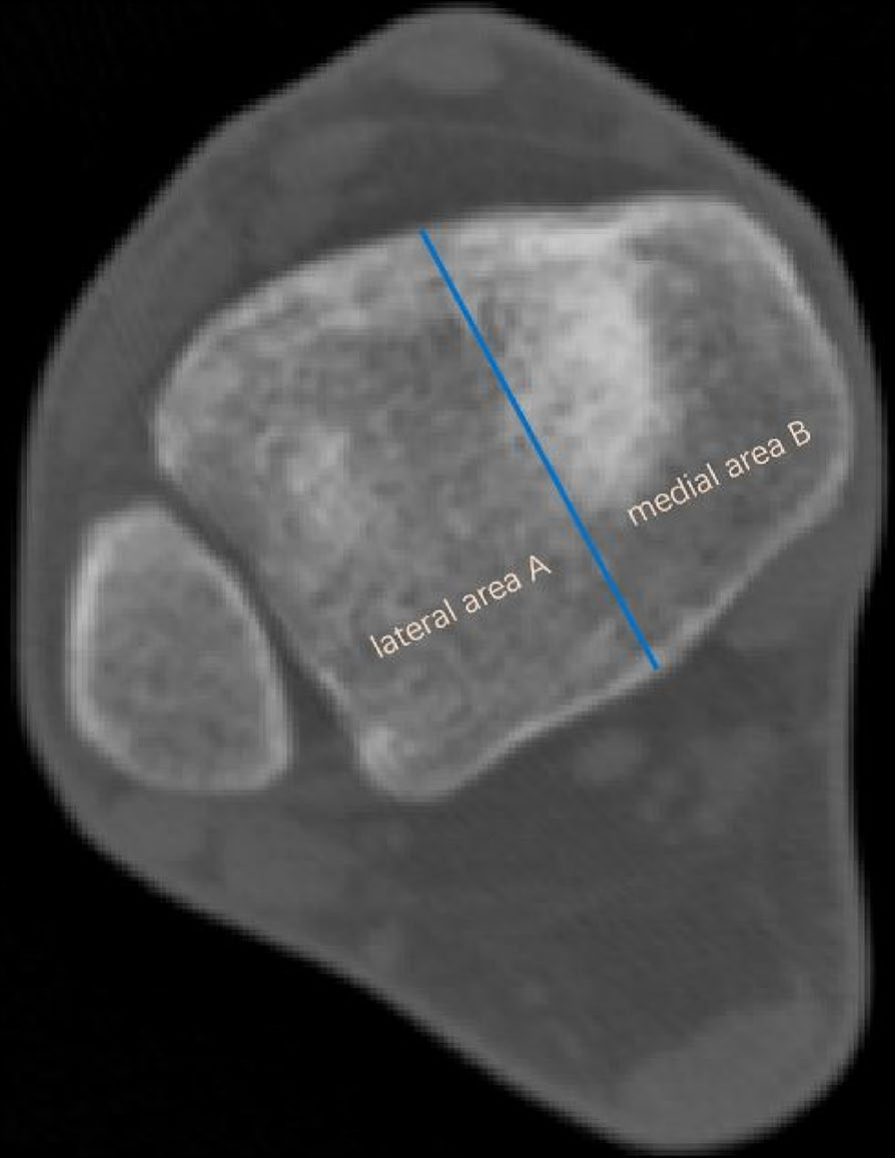
Division of the tibial plafond: lateral area A and medial area B

To study the location of IAIF, we divided the posterior part of the tibial plafond into two parts by the midpoint of the posterior tibial edge and named the lateral area A and the medial area B (Fig. 3).

### Data analysis

Patient characteristics were summarized as proportions and mean, with the range given in parentheses. Statistical analysis was performed using SPSS V23.0 (Chicago, IL, USA). The 108 cases were divided into two groups; IAIF and NIAIF (no intraarticular impacted fragments). Univariate binary logistic analysis was used to analyze the related factors such as age (> 60 years, ≤ 60 years), gender, involvement of medial malleolus (fracture line extended to medial malleolus), involvement of fibular notch, ratio of fibular notch, length of major posterior malleolar fracture fragment, area of posterior malleolar fracture fragment, Haraguchi classification, and Mason classification between group IAIF and group NIAIF. Spearman’s correlation was performed to determine the correlation among the area of IAIF and other factors. Statistical significance was declared for *P* < 0.05.

## Results

According to the Lauge–Hansen classification system, 108 cases were classified as follows: 8 (7.4%) pronation-abduction (PAB), 15 (13.9%) pronation-external rotation (PER), and 85 (78.7%) supination-external rotation (SER). For the OTA/AO system, consensus classification demonstrated 86 (85.1%) as B and 15 (14.9%) as C. As for the Haraguchi classification, we determined that 71 (65.7%) were type 1, 25 (23.1%) type 2, and 12 (11.2%) type 3. With the Mason classification, consensus classification demonstrated 12 (11.2%) as type 1, 71 (65.7%) as type 2A, 22 (20.3%) as type 2B, and 3 (2.8%) as type 3. Of the 108 cases, we also compared the fracture classifications between cases with IAIF (75, 69.4%) and those without (33, 30.6%) (Table 1).

Among the 108 cases, the fibular notch was involved in 89 (82.4%) cases. Average *L*_IFN_/(*L*_IFN_ + *L*_SFN_) was 32.9% (11.6–64.9%); average *L*_MPMF_ and *L*_IAIF_ were 8.07 mm (0.8–25.74 mm) and 6.17 mm (2.09–12.72 mm), respectively. The average *H*_IAIF_ was 2.91 mm (0.72–9.35 mm). The average *A*_PMF_ was 172.64 mm^2^ (12.52–523.71 mm^2^), that of *A*_IAIF_ was 60.01 mm^2^ (4.87–199.94 mm^2^), and *A*_STP_ was 1101.28 mm^2^ (676.47–1701.83 mm^2^). Therefore, *L*_MPMF_/(*L*_MPMF_ + *L*_STP_) was 20.8% (3.0–63.7%) on average. *A*_PMF_/(*A*_PMF_ + *A*_STP_ + *A*_IAIF_) was 13.1% (0.8–39.7%) on average, that of *A*_IAIF_/(*A*_PMF_ + *A*_STP_ + *A*_IAIF_) was 4.7% (0.4–17.8%) and *A*_IAIF_/*A*_PMF_ was 52.6% (1.2–235.4%). There were 25 (23.1%) cases of posterior malleolar fractures involving medial malleolus. The area of posteromedial fragments and posterolateral fragments of 23 (21.3%) cases were measured separately. The average ratio of the posteromedial fragments to posterolateral fragments was 89.8% (8.75–189.6%) (Table 2).

**Table 2:** Related measurement of posterior malleolar fractures

	Length (mm)	Area (mm^2^)	Ratio (%)
*L*_MPMF_	8.07 (0.8–25.74)		
IAIF	6.17 (2.09–12.72)	60.01 (4.87–199.94)	
*H*_IAIF_	2.91 (0.72–9.35)		
*A*_PMF_		172.64 (12.52–523.71)	
*L*_IFN_	9.44 (3.05–20.09)		
*L*_IFN_/(*L*_IFN_ + *L*_SFN_)			32.9 (11.6–64.9)
*L*_MPMF_/(*L*_MPMF_ + *L*_STP_)			20.8 (3.0–63.7)
*A*_PMF_/(*A*_PMF_ + *A*_STP_ + *A*_IAIF_)			13.1 (0.8–39.7)
*A*_IAIF_/(*A*_PMF_ + *A*_STP_ + *A*_IAIF_)			4.7 (0.4–17.8)
*A*_IAIF_/*A*_PMF_			52.6 (1.2–235.4)

Values are given as the mean, with the range in parentheses

As for the location of IAIF, there were 75 (69.4%) cases with IAIF. Sixty-five cases with single IAIF were distributed into 43 (57.3%) cases in area A, 16 (21.3%) cases in area B, and 6 (8%) cases in the dividing line of area A and B (A&B). In the nine cases with two IAIFs, one (1.3%) case was located in area A and eight (10.7%) cases were located in both area A and B. There was one (1.3%) case with three IAIFs, two of which were located in area A, with another one in area B (Table 3).

**Table 3:** The numbers and location of IAIF

IAIF numbers	IAIF location		
	A (%)	B (%)	A&B (%)
1	43 (50%)	16 (18.6%)	6 (7%)
2	10 (11.6%)	8 (9.3%)	0
3	2 (2.3%)	1 (1.2%)	0

*IAIF* intraarticular impacted fragments

IAIF occurrence was significantly related with the involvement of medial malleolus (fracture line extended to medial malleolus, *P* = 0.022), the involvement of fibular notch (*P* = 0.021), *L*_IFN_/(*L*_IFN_ + *L*_SFN_) (*P* = 0.037), *L*_MPMF_ (*P* = 0.004), *A*_PMF_ (*P* = 0.010), Haraguchi classification (*P* = 0.037), and Mason classification (*P* = 0.038). There was no significant difference in the intragroup comparison of Haraguchi and Mason classification (Table 4). We further explored the correlation between *A*_IAIF_ and *A*_PMF_, *L*_MPMF_, *L*_IFN_, and age. The result showed that the *A*_IAIF_ was irrelevant to the factors mentioned above (Table 5).

**Table 4:** Univariate analysis results for the relation of IAIF

Variables	IAIF (*n* = 75)	NIAIF (*n* = 33)	Statistical values	*P*-Value
Age (> 60 years/ ≤ 60 years)	20/55	12/21	1.033	0.309^a^
Gender (Male/Female)	20/55	14/19	2.638	0.104^a^
Involvement of medial malleolus	22/53	3/30	5.278	0.022^a^
Involvement of fibular notch	66/9	23/10	5.295	0.021^a^
*L*_IFN_/(*L*_IFN_ + *L*_SFN_)*	0.27 ± 0.13	0.25 ± 0.13	−2.115	0.037^b^
*L*_MPMF_^#^	7.30 (1.89–25.74)	5.40 (0.80–16.10)	−2.868	0.004^c^
*A*_PMF_^#^	147.15(12.96–523.71)	95.60 (12.52–466.17)	−2.438	0.010^c^
Haraguchi (1/2/3)^§^			6.569	0.037^a^
1	48	24		
2	21	3		
3	6	6		
Mason (1/2A/2B/3)^§^			7.886	0.038^a^
1	6	6		
2A	47	24		
2B	20	2		
3	2	1		

*IAIF* intraarticular impacted fragments, *NIAIF* no intraarticular impacted fragments

^*^Values are given as the mean ± SD

^#^Values are given as the median, with the range in parentheses

^§^Chi-square test of Haraguchi (1/2/3) (*P* = 0.037) and Mason (1/2A/2B/3) (*P* = 0.038). By pairwise comparison, there was no significant difference among the intragroup comparison in Haraguchi (1/2/3) and Mason (1/2A/2B/3) through Fisher’s exact test

^a^Chi-square test

^b^*t*-test

^c^Mann–Whitney *U* test

**Table 5:** Spearman correlation analysis for area of IAIF

	RS	*P*-Value^*^
*A*_IAIF_ and *A*_PMF_	−0.41	0.729
*A*_IAIF_ and *L*_MPMF_	−0.165	0.157
*A*_IAIF_ and *L*_IFN_	−0.230	0.063
*A*_IAIF_ and age	−0.110	0.348

^*^P < 0.05 assumed statistical significance, based on Spearman correlation analysis

## Discussion

There was a high incidence of IAIF in posterior malleolar fractures. Through the morphological analysis of posterior malleolar fractures on CT scans, 75 cases (69.4%) of posterior malleolar fractures presented with IAIF, which was far beyond our expectations. CT scans are beneficial in identifying IAIF since most IAIF cannot be found by X-ray. More attentions should be paid to the size of the posterior malleolar fragments and its effect on ankle stability and contact stress [20–22].

In our study, 71 (65.7%) cases of posterior malleolar fractures with single fragment could be classified as Haraguchi 1 or Mason 2A. IAIF was found in more than 2/3 cases of single fragment fractures, and the average area of fragments was larger than those without IAIF. Haraguchi et al. [18] claimed type 1 was a posterior malleolar avulsion fracture, while Mason et al. [19] indicated type 2A was a “push-off” fracture (the loaded talus pushes off the posterolateral corner of the tibia). Due to the high incidence of IAIF in the single fragment, we speculate that the mechanisms of injury of single fragment fracture may include rotation impact, and not just avulsion fracture. The larger the posterior malleolar fragments, the higher the proportion of rotation impact.

There were 22 (20.4%) cases of posterior malleolar fractures with multifragment that could be regarded as Haraguchi 2 or Mason 2B. Among the fractures, 20 cases (90.9%) were found with IAIF, and 17 cases (77.3%) of IAIF were distributed in medial area B (deep of posteromedial fragment). Mason et al. [19] believed the mechanism of Mason 2B was that the loaded talus pushes off the posterolateral corner of the tibia when rotated in the ankle mortise and with continued rotation, the posteromedial corner was also fractured as a separate fragment. Vosoughi et al. [23] studied Mason 2B, in which separate posteromedial fragment was associated with a posterolateral fragment in a rotational injury that included both supination external rotation (SER) and pronation external rotation (PER) injuries. Gardner et al. [24] supposed external rotation and hyperplantarflexion as the mechanism of multifragment. IAIF in multifragment fractures was primarily located in medial area B. We suppose the mechanism of multifragment fractures is consistent with the view of Mason.

An unexpected result was that 50% Haraguchi 3 fractures accompanied IAIF in 12 (11.2%) cases of Haraguchi 3 (Mason 1) fractures. The Haraguchi 3 fracture is an inferior transverse tibiofibular ligament (deep fibers of posterior inferior tibiofibular ligament) avulsion fracture [18, 19], not as small as “bone chips.” We believe it has been significantly underestimated in the current literature. Some IAIFs can be found in Haraguchi 3 fractures and can only be observed on CT scans. Attention should be paid to the posterior malleolar fractures, even the Haraguchi 3 fractures.

Almost all posterior malleolar fractures of Haraguchi 2 or Mason 2B involve the medial malleolus and fibular notch [18, 19]. Through univariate analysis, it was found that the involvement of medial malleolus (fracture line extended to medial malleolus, *P* = 0.022) was related to the incidence of IAIF. The fibular notch is very important for the stability of the ankle joints [25]. Fracture lines in 25 (23.1%) cases in which the medial malleolus was involved had extended to the fibular notch. By univariate analysis, it was found that the involvement of fibular notch (*P* = 0.021) was also related to the incidence of IAIF. A total of 89 (82.4%) cases involved fibular notch, and the *L*_IFN_/(*L*_IFN_ + *L*_SFN_) was 26.7% (9.4–62.9%) on average. Greater involvement of fibular notch and bigger posterior malleolar fragments are associated with higher incidence of IAIF.

Related measurement and calculation of the posterior malleolar fractures showed that the *A*_IAIF_ was 60.01 mm^2^ (4.87–199.94 mm^2^) on average, and the *A*_IAIF_/*A*_PMF_ was 52.6% (1.2–235.4%). With an average ratio over 50%, some IAIF are larger than posterior malleolar fragments. No correlation between the *A*_IAIF_ and other factors, such as *A*_PMF_, *L*_MPMF_, *L*_IFN_, and age were found. In our study, some small posterior malleolar fragments with very large IAIF were identified, so IAIF should not be ignored in posterior malleolar fractures.

For single fragment fractures (Mason 2A), we choose a posterolateral approach to expose posterior malleolar fragment that needs to be elevated proximally to distally, and then IAIF can be identified and reduced. Finally, posterior malleolar fragment is reduced anatomically, covering IAIF, and fixed with screws. In multifragment fractures (Mason 2B), a posterolateral approach combined with posteromedial approach and modified posteromedial approach are both options [26, 27]. A modified posteromedial approach is preferred in our institution. Both posterolateral and posteromedial fragments can be exposed through this single straight approach, and posterolateral and posteromedial fragments are elevated from interfragment fracture line to laterally and medially, respectively, named as “opening book.” IAIFs in lateral or medial side can then be found and reduced.

There are several limitations in this study. First, the small cohort of patients may affect the accuracy of the results, more patients are required for future research. Second, only the surgery patients are included because CT scans were seldom performed on patients with nondisplaced ankle fractures in our institution. Third, we use the techniques of Mimics Software to measure length and area manually, which are subjective and the results are mainly based on descriptions.

In conclusion, our research indicates a high incidence of IAIF in posterior malleolar fractures. We describe the morphology of posterior malleolar fragments, as well as IAIF, in detail. All IAIFs are found in the posterior malleolar, and the most common location is within the lateral area A. It heralds the incidence of IAIF when posterior malleolar fracture lines extend to medial malleolus or fibular notch. *L*_IFN_/(*L*_IFN_ + *L*_SFN_), *L*_MPMF_ and *A*_PMF_ are also associated with the incidence of IAIF. IAIF is also helpful to understand the mechanism of posterior malleolar fractures. Our studies indicate that CT scans are most effective for ankle fractures with posterior malleolar fractures in determining the occurrence of IAIF and making the operation plan. The choice of a surgical approach is mainly based on the morphology of posterior malleolar fragments and the location of IAIF.

## Data Availability

The datasets used and/or analyzed during the current study are available from the corresponding author on reasonable request.

## Abbreviations

IAIF: Intraarticular impacted fragment
*L*_IFN_: Length of involved fibular notch
*L*_SFN_: Length of stable fibular notch
*L*_IAIF_: Length of IAIF
*A*_IAIF_: Area of IAIF
*A*_PMF_: Area of posterior malleolar fragment
*A*_STP_: Area of stable tibial plafond
*L*_MPMF_: Length of major posterior malleolar fragment
*L*_STP_: Length of stable tibial plafond
*H*_IAIF_: Height of IAIF
PAB: Pronation-abduction
PER: Pronation-external rotation
SER: Supination-external rotation
CT: Computed tomography
